# *ins-7* Gene Expression Is Partially Regulated by the DAF-16/IIS Signaling Pathway in *Caenorhabditis elegans* under Celecoxib Intervention

**DOI:** 10.1371/journal.pone.0100320

**Published:** 2014-06-19

**Authors:** Shanqing Zheng, Sentai Liao, Yuxiao Zou, Zhi Qu, Fan Liu

**Affiliations:** Sericulture & Agri-Food Research Institute, Guangdong Academy of Agricultural Sciences, Guangzhou, Guangdong, China; CSIR-Central Drug Research Institute, India

## Abstract

DAF-16 target genes are employed as reporters of the insulin/IGF-1 like signal pathway (IIS), and this is notably true when *Caenorhabditis elegans* (*C. elegans*) is used to study the action of anti-aging compounds on IIS activity. However, some of these genes may not be specific to DAF-16, even if their expression levels are altered when DAF-16 is activated. Celecoxib was reported to extend the lifespan of *C. elegans* through activation of DAF-16. Our results confirmed the function of celecoxib on aging; however, we found that the expression of *ins-7*, a DAF-16 target gene, was abnormally regulated by celecoxib. *ins-7* plays an important role in regulating aging, and its expression is suppressed in *C. elegans* when DAF-16 is activated. However, we found that celecoxib upregulated the expression of *ins-7* in contrast to its role in DAF-16 activation. Our subsequent analysis indicated that the expression level of *ins-7* in *C. elegans* was negatively regulated by DAF-16 activity. Additionally, its expression was also positively regulated by DAF-16-independent mechanisms, at least following external pharmacological intervention. Our study suggests that *ins-7* is not a specific target gene of DAF-16, and should not be chosen as a reporter for IIS activity. This conclusion is important in the study of INSs on aging in *C. elegans*, especially under the circumstance of drug intervention.

## Introduction

From invertebrates to vertebrates, the insulin/IGF-1-like signaling pathway (IIS) is evolutionary conservation and plays an important role in regulating animal development and pathology [1]–[4]. In vertebrates, phosphatidylinositol 3-kinase (PI3K) is activated to generate phosphatidylinositol 3, 4, 5-triphosphate (PIP3) when the cell membrane-localized IIS receptor is stimulated by insulin or IGF-1. Subsequently, protein kinase B (PKB) is localized to the cell membrane by PIP3 and activated by PDK-1/2 to phosphorylate FOXO transcription factors [5]. In *C. elegans*, insulin-like proteins activate the PI3K homolog AGE-1 through the IIS receptor DAF-2, ultimately directing the AKT-1/2 and SGK-1 kinases to phosphorylate the FOXO protein DAF-16 with phosphorylated DAF-16 accumulating in the nuclei [6]. The IIS signaling pathway has been shown to regulate dauer formation, improve heat and oxidative stress resistance, extend lifespan, and delay the onset of many age-related diseases in *C. elegans* [7]–[11]. Decreased IIS signal transduction releases the FOXO protein DAF-16 into the nucleus to regulate the expression levels of many genes.

Many DAF-16 target genes have been reported to control aging in *C. elegans* [6]. *ins-7*, one of about 40 insulin-like genes that have been identified in the *C. elegans* genome [12], [13], is downregulated in *daf-2* loss-of-function mutants and upregulated in *daf-16* null mutants [6]. Many INS proteins are present in the nervous system of *C. elegans*, and killing these sensory neurons or inhibiting their functions triggers DAF-16 nuclear localization [14]–[16]. However, to the best of our knowledge, the functions of the 40 INS proteins in *C. elegans* have not been well characterized. Wild-type (N2) worms treated with *ins-7 *RNAi have an extended lifespan, and *ins-7* RNAi also results in increased nuclear accumulation of DAF-16::GFP in intestine cells [16]. Murphy *et al.* reported that DAF-2 and DAF-16 regulate *ins-7* expression in the intestine and that downregulation of *ins-7* expression in the intestine lowers INS-7 levels in other tissues, which in turn triggers DAF-16 activity in the muscles and hypodermis [16]. It has also been reported that *ins-7* expression in URX neurons can regulate aversive olfactory learning by antagonizing DAF-2, the receptor of the IIS signaling pathway [17]. Together, these studies reveal that the expression of *ins-7* is regulated by the DAF-16/IIS signal pathway and that *ins-7* can also influence the function of IIS by means of its feedback regulation of DAF-2 [16].

*C. elegans* has been widely used as a model to screen for anti-aging compounds and to study the mechanisms of aging. Many compounds have been reported to have effects on the aging of *C. elegans* [18]. A well-characterized compound with an influence on aging would be a valuable tool/drug to study the mechanisms of aging and investigate how endogenous systems change with aging. We used celecoxib as our experimental compound due to its established effect on the aging of *C. elegans.* Celecoxib extends the lifespan of *C. elegans* by activating DAF-16 and subsequently alters the expression of DAF-16 target genes [19]. According to our study, celecoxib extended the lifespan of *C. elegans* via DAF-16 activity and regulated the expression levels of DAF-16 target genes: *scl-20*, *K09F6.6*, *sod-3*, and *ins-7*. Interestingly, the expression of *ins-7*, which is typically downregulated when DAF-16 is activated, was significantly upregulated in celecoxib-treated N2 worms. Our results also showed that *ins-7* expression was upregulated even in *daf-16* and *daf-2;daf-16* double mutants following treatment with celecoxib. Consequently, our work indicated that the expression of *ins-7* was negatively regulated by DAF-16 activity, but that it could also be positively regulated by other DAF-16–independent mechanisms, at least under external pharmacological intervention. We confirmed that *ins-7* was not a specific target gene of DAF-16 and also expanded our understanding of the regulation of *ins-7* expression, further supporting its importance in the study of aging and the function of insulin-like genes.

## Materials and Methods

### Strains

All strains used in this work were provided by the Caenorhabditis Genetics Center and maintained and handled according to standard protocols as described previously [20]. Strains used in this study were: N2, Bristol (wild-type); CF1442, *daf-2(e1370) III*; *daf-16 (mu86) I*; DR1572, *daf-2(e1368) III*; CF1038, *daf-16(mu86) I*; CB1370, *daf-2(e1370) III*; RB1388, *ins-7* (ok1573) IV, HT1702, *unc-119(ed3) III*; wwEx66 [ins-7p::GFP+unc-119(+)]; TJ356, zIs356 [*Pdaf-16::daf-16-gfp;rol-6*] IV; UL1735 (*Ppqm-1::gfp*) and RB711, *pqm-1(ok485)*.

### Lifespan Tests

Lifespan assays were performed on nematode growth media (NGM) plates. Synchronized L4 or young adult worms were transferred to 5 NGM plates (6 cm diameter). A total of 20–30 worms were transferred on to each NGM plate containing 40 µM FUDR. All lifespan assays were performed at 20°C. The worms were scored as live, dead, or lost and transferred to new NGM plates every other day. Worms that failed to display touch-provoked movement were scored as dead. For celecoxib treatment, 500 µL of 10 µM celecoxib (Sigma-Aldrich, St. Louis, MO, USA) was added on to the surface of NGM media 8 hours before transferring worms onto the plates. Celecoxib was dissolved in DMSO for storage and diluted in water to work concentration before use. After adding the compound to the NGM plates, the final DMSO concentration was 0.1%. Control plates contained 0.1% DMSO too. All lifespan tests were repeated three times. Survival curves and statistical analyses were carried out using SPSS software (SPSS, Chicago, IL, USA). P values were calculated using the log-rank test.

### Quantitative Real-time PCR Experiments

Young adult N2 worms were transferred on to control plates or those containing 10 µM celecoxib, cultured at 20°C, and harvested after 24 hours, 7 days, or 14 days. Total RNA was isolated from about 2000 worms using an RNA isolation kit (RNAiso Plus; TaKaRa Bio, Shiga, Japan). A total of 5 µg RNA was used to prepare cDNA using a High Capacity cDNA Reverse Transcription Kit (Applied Biosystems, Foster City, CA, USA). The real-time PCR reactions were performed using Power SYBR Green PCR Master Mix and an ABI 7500 system (Applied Biosystems, Foster City, CA, USA). The relative expression levels of genes were determined using the 2^−ΔΔCT^ method and normalized to *cdc-42* and *act-1* [21]–[23]. Primer sequences used are summarized in supplement information (Table S6).

### RNAi Knockdown of Gene Expression

An RNAi bacterial strain (HT115) expressing a double-stranded *daf-16* RNA (vector, L4440) was cultured and used to inactivate *daf-16* function. The identity of the clones was confirmed by sequencing. Eggs from HT1702 or UL1735 worms were transferred to fresh NGM plates containing *daf-16* RNAi bacteria and allowed to grow at 15°C for 3 days. Larva stage 4 HT1702 or UL1735 worms were then transferred to the same RNAi NGM plates in the presence or absence of 10 µM celecoxib and cultured at 20°C for 24 hours. The RNAi NGM plates contained 1 mM isopropyl-B-D-thiogalactopyranoside (IPTG) for the induction of double stranded RNA [19], [24].

### GFP Fluorescent Analysis

After the HT1702 worms were cultured on RNAi NGM plates, about 20 control or celecoxib-treated worms were mounted on 1% agarose slides (10–20 per slide). The induction of INS-7::GFP in the body of worms was assayed using a Ti fluorescent microscope (Nikon, Tokyo, Japan). The average GFP intensity was calculated using the Metamorph software package (Molecular Devices, Sunnyvale, CA, USA).

GFP nuclear localization was analyzed as described previously [19]. Young adult worms (TJ356, UL1735 or UL1735;*daf-16 RNAi*) were seeded on to either control or celecoxib plates and cultured at 20°C for 24 hours. GFP expression was then analyzed using a Ti fluorescent microscope (Nikon, Tokyo, Japan) at 10× or 40× magnification. Worms were scored for the presence or absence of GFP accumulation. Animals were scored as having nuclear GFP if more than one intestinal nuclei contained GFP.

## Results

### Celecoxib Extends the Lifespan of *C. elegans* via DAF-16

**Figure 1:**
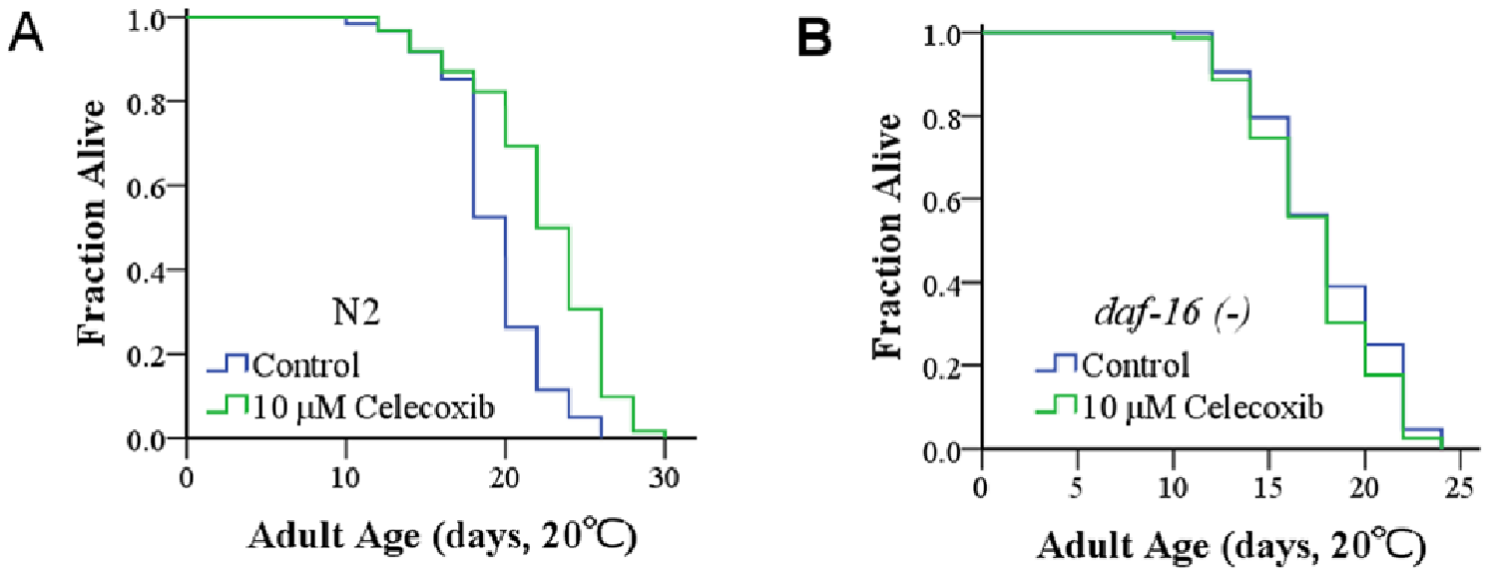
Celecoxib extends the lifespan of *C. elegans* via DAF-16. Synchronized young adult N2 worms (**A**) or *daf-16* (**−**) worms (**B**) were cultured on NGM plates in the presence or absence of 10 µM celecoxib. Mean lifespan of N2 worms treated with 10 µM celecoxib was significantly longer than that of control worms. Celecoxib had no effect on aging of *daf-16* (**−**) mutants. One representative result from three independent experiments is presented. Survival curves were generated using SPSS software (SPSS, Chicago, IL, USA). P values and mean lifespans were calculated using a log-rank test (Table 1).

We used celecoxib to study the pharmacological intervention of aging on *C. elegans*. According to previous studies, celecoxib extends the lifespan of *C. elegans* by decreasing IIS signal transduction, which serves to activate DAF-16 [19]. We treated young adult N2 worms with 10 µM celecoxib and found that the mean lifespan of N2 worms was extended by up to 18% at 20°C (mean increase of three independent experiments) (Figure 1A, Table 1). We also treated *daf-16* null-mutant worms with 10 µM celecoxib. Our results indicated that celecoxib had no effect on the lifespan of *daf-16 (mu86) I* worms (Figure 1B, Table 1). These results were consistent with previous studies indicating that celecoxib extended the lifespan of *C. elegans* by way of DAF-16 activity.

**Table 1:** The effects of celecoxib on lifespan.

Experiment	Strain	Condition	Deaths	Mean lifespan ± SEM	Increase (%)	P value
**1**	N2	Control	87	17.04±0.42		
		10 µM celecoxib	90	19.66±0.50	15.38	0.005
	*daf-16* (**−**)	Control	77	15.72±0.26		
		10 µM celecoxib	86	15.45±0.76	#	0.984
	*ins-7*(**−**)	Control	70	19.08±0.29		
		10 µM celecoxib	69	22.89±0.76	19.96	<0.001
**2**	N2	Control	94	17.85±0.43		
		10 µM celecoxib	102	21.51±0.67	20.5	<0.001
	*daf-16* (**−**)	Control	78	14.98±0.33		
		10 µM celecoxib	84	14.31±0.24	#	0.752
	*ins-7*(**−**)	Control	91	19.92±1.02		
		10 µM celecoxib	93	24.13±0.94	21.13	<0.001
**3**	N2	Control	110	18.18±0.78		
		10 µM celecoxib	121	22.00±0.52	21.01	<0.001
	*daf-16* (**−**)	Control	99	15.04±0.38		
		10 µM celecoxib	85	14.87±0.19	#	0.623
	*ins-7*(**−**)	Control	85	18.56±0.87		
		10 µM celecoxib	101	23.14±0.93	24.68	<0.001
**Sum of three experiments**			**Total**	**Mean increase (%)**		
	N2	Control	291			
		10 µM celecoxib	313	18.96		
	*daf-16* (**−**)	Control	254			
		10 µM celecoxib	255	#		
	*ins-7*(**−**)	Control	246			
		10 µM celecoxib	263	21.92		

P values were calculated for individual experiments, each including control and experimental animals and performed at the same time. The table shows the number of dead animals. Animals that crawled off the plate, bagged, or burst were censored and were therefore excluded from all analysis. All statistics were calculated using SPSS software (SPSS, Chicago, IL, USA). The log rank (Mantel-Cox) test was used for statistical analysis. #no significant different (P>0.05).

### The Expression of *ins-7* in N2 Worms is Positively Regulated by Celecoxib

**Figure 2:**
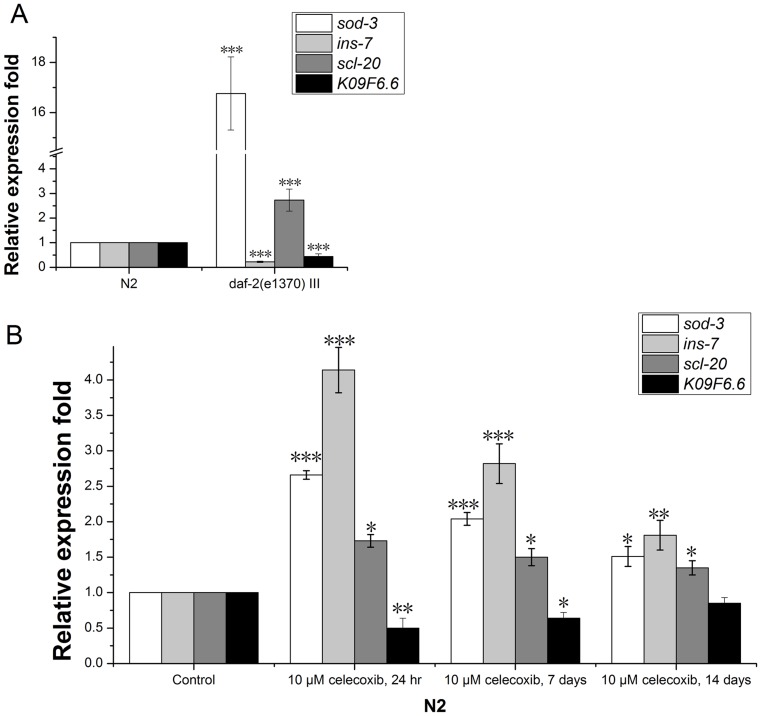
Celecoxib regulates DAF-16 target gene expression. *scl-20, K09F6.6, ins-7*, and *sod-3* expression profiles in *daf-2 (e1370) III* mutants compared to N2 worms (**A**). *ins-7* was significantly upregulated in N2 worms treated with 10 µM celecoxib (**B**). Celecoxib was previously reported to regulate DAF-16 target genes by decreasing IIS signal transduction activity. *ins-7* was reported to be a target gene of DAF-16 and downregulated when IIS signal activity was decreased. These results indicated *ins-7* was uncharacteristically upregulated in celecoxib-treated N2 worms. The results of at least three independent experiments are presented. Data are averages of real-time PCR results ± Standard Deviation (SD). Error bars represent SD. P values were calculated by using a T-test, ***P<0.001. Also see Table S2.

*sod-3* has been reported to be a specific target gene of DAF-16, and its expression level is upregulated when DAF-16 is activated [25]–[28]. *ins-7* has been reported to be downregulated in *daf-2* mutants or when DAF-16 is activated [6]. We used *daf-2 (e1370) III* to test the expression of *sod-3* and *ins-7*. According to our results, the expression profiles of *sod-3* and *ins-7* were consistent with these previous studies (Figure 2A, Table S1). In order to confirm that celecoxib extends the lifespan of *C. elegans* by activating DAF-16, we tested the expression of DAF-16 target genes in celecoxib-treated worms. About 2000 young adult N2 worms were cultured on NGM plates containing 10 µM celecoxib or control plates for 24 hours, 7 days, or 14 days. Total RNA was then isolated from these worms. The relative expression of *sod-3* and *ins-7* were calculated using real-time PCR. Our results showed that *sod-3* expression was significantly upregulated in celecoxib-treated N2 worms (Figure 2B, Table S2), this result was consistent with previous reports [19]. Celecoxib extends the lifespan of *C. elegans* by decreasing IIS signal transduction to activate DAF-16, so celecoxib-treated N2 worms should have lower the expression levels of *ins-7* according to previous reports [6], [16]. However, our results indicated that *ins-7* was significantly upregulated following celecoxib treatment (Figure 2B, Table S2). Furthermore, we also observed that the mean lifespan of *ins-7* mutant worms treated with celecoxib was increased by up to 21.92% (mean increase of three independent experiments; Table 1), a greater increase when compared to celecoxib-treatment alone. This discrepancy led us to speculate that there were other mechanisms regulating *ins-7* expression in *C. elegans* following celecoxib intervention.

IIS regulates two classes of genes through DAF-16. Class I genes are upregulated when the IIS signal is reduced or DAF-16 is translocated to the nuclei. Class II genes display the opposite profile [6]. There are two elements in the promoters of DAF-16 targets: a DAF-16 binding element (DBE) and a DAF-16 associated element (DAE). According to the previous studies, both DAE and DBE are present in the promoters of both gene classes and DAF-16 directly regulates the expression of class I genes through DBE [29], [30]. The class II genes may be regulated by other co-factors in addition to DAF-16. *sod-3* is a class I gene and only contains a DBE. *ins-7* is a class II gene and only contains a DAE. Our results suggested that *ins-7* was positively regulated by celecoxib, so we also tested the effects of celecoxib on the expression of *scl-20* (class I, DAE only) and *K09F6.6* (class II, DBE only). We found that expression of *scl-20* was upregulated and that of *K09F6.6* was downregulated following treatment with celecoxib in N2 worms (Figure 2B, Table S2). These expression profiles were consistent with the altered activity of DAF-16 following celecoxib treatment. Together, these results led us to speculate that *ins-7* was not directly regulated by DAF-16.

### *ins-7* Expression Partially Depends on DAF-16/IIS Activity

**Figure 3:**
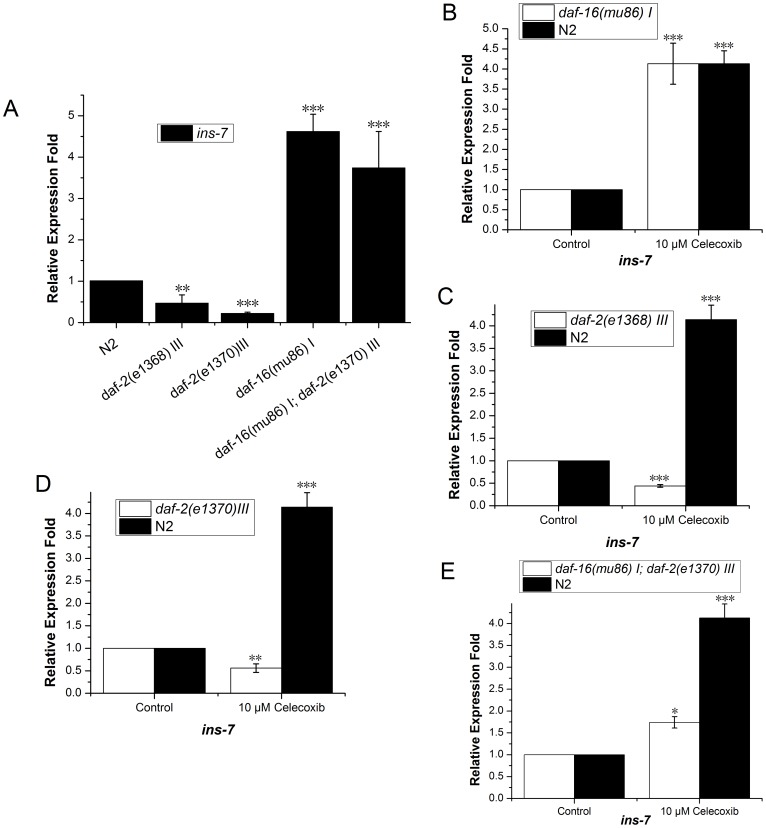
*ins-7* expression patterns in *daf-2* and *daf-16* mutant worms with or without celecoxib treatment. *ins-7* was downregulated in *daf-2 (e1370) III* mutants and upregulated in *daf-16 (mu86) I* mutants and *daf-*2 *(e1370) III*; *daf-16 (mu86) I* double mutants (**A**). *ins-7* was significantly upregulated in *daf-16 (mu86) I* worms treated with 10 µM celecoxib (**B**). *ins-7* was significantly downregulated in *daf-2 (e1370) III* and *daf-2 (e1368) III* worms treated with 10 µM celecoxib (**C, D**). *ins-7* expression was still significantly upregulated in *daf-*2*(e1370) III*; *daf-16 (mu86) I* double mutants treated with 10 µM celecoxib (**E**). The results of at least three independent experiments are presented. Data are averages of real-time PCR results ± SD. Error bars represent SD. P values were calculated using a T-test, *P<0.05; **P<0.01; ***P<0.001. Also see Table S1 and S3.

INS-7, an insulin-like protein, is reported to be a possible DAF-2 agonist and negatively regulated by DAF-16 activity [6]. It has also been reported that *ins-7* might regulate DAF-16 activity by way of feedback regulation of DAF-2 [16]. We tested *ins-7* expression levels in *daf-2(e1368) III*, *daf-2(e1370) III*, and *daf-16 (mu86) I* mutants. The results were consistent with previous reports and indicated that *ins-7* expression is downregulated in *daf-2* mutants and upregulated in *daf-16* mutants (Figure 2A and 3A, Table S1).

According to our results, *ins-7* was upregulated approximately 4-fold following celecoxib treatment of N2 worms (Figure 2B, Table S2), which was in contrast to its expression pattern when DAF-16 was activated. If *ins-7* expression is specifically related to DAF-16 activity, its expression in celecoxib-treated *daf-16 (* **−** *)* mutant worms should demonstrate no significant change compared to controls. However, we found that the expression of *ins-7* was significantly upregulated in *daf-16 (* **−** *)* worms when treated with celecoxib (Figure 3B, Table S3). This result suggested that *ins-7* expression was not specific to DAF-16. When we treated *daf-2* mutant worms with celecoxib, we found *ins-7* expression was downregulated about 50% compared to controls (Figure 3C and D, Table S3). This result was consistent with the idea that celecoxib negatively regulates IIS signal transduction by decreasing PDK-1 activity, as the expression of *ins-7* was downregulated when IIS signal transduction was decreased [16], [19]. The above results led us to speculate that *ins-7* gene expression in N2 worms could be regulated by some other mechanisms in addition to being regulated by IIS and DAF-16. In order to test this hypothesis, we analyzed *ins-7* expression in *daf-2; daf-16* double mutants treated with celecoxib. The resulting expression level of *ins-7* was upregulated by up to 70% (Figure 3E, Table S3). This result was consistent with the idea that *ins-7* expression could be positively regulated by other pathways independent or partially independent of IIS activity.

### *ins-7* is Partially Regulated by PQM-1 Activity

**Figure 4:**
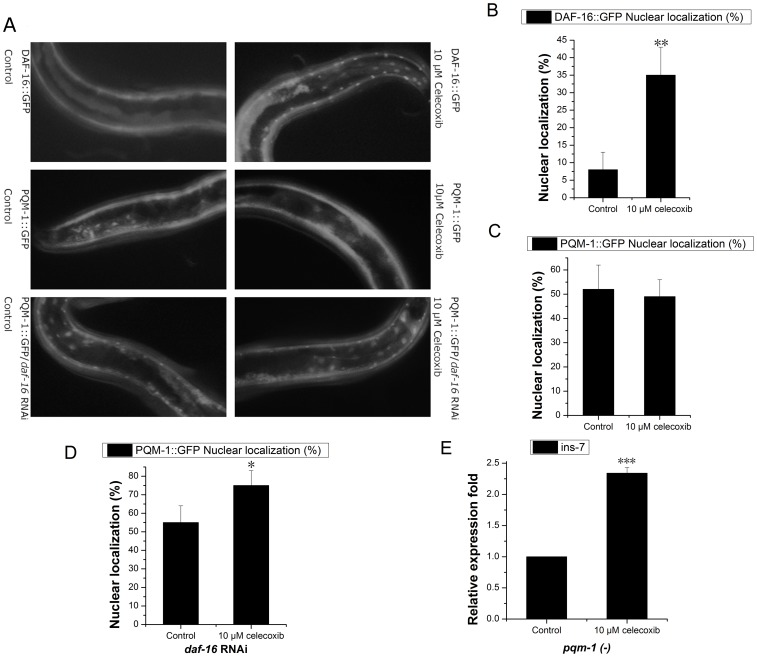
The effects of celecoxib on DAF-16::GFP and PQM-1::GFP nuclear localization. Celecoxib enhanced DAF-16 nuclear localization (**A, B**) and PQM-1::GFP nuclear localization in *daf-16 (RNAi)* worms (**A, C**). However, PQM-1::GFP nuclear localization was not significantly changed following celecoxib treatment in N2 worms (**A, D**). *ins-7* was upregulated following celecoxib treatment in *pqm-1* mutants (**E**). Error bars represent SD. P values were calculated using a T-test, *P<0.05; **P<0.01; ***P<0.001. Also see Table S2 and S4.

It has been reported that the transcription factor PQM-1 is strongly dependent on IIS activity and directly controls class II gene expression via binding to DAE motifs [6], [30]. We tested the effect of celecoxib on *pqm-1* expression. Our results showed that celecoxib had no effect on the expression level of *pqm-1* in N2 worms. IIS regulation of PQM-1 is modulated primarily through posttranslational regulation of its subcellular localization [30]. We found that 10 µM celecoxib could enhance DAF-16 nuclear translocation (Figure 4A and B, Table S4). This result is consist with a previous report [19], but we found that celecoxib had no obvious function on PQM-1::GFP nuclear translocation (Figure 4A and C, Table S4). The nuclear localization of PQM-1::GFP is opposite of that of DAF-16::GFP [30], and when we treated PQM-1::GFP worms with *daf-16* RNAi we found that the PQM-1::GFP nuclear location was further enhanced by celecoxib (Figure 4A and D, Table S4). According to this result, we speculated that *ins-7* was partially regulated by PQM-1 in celecoxib-treated N2 worms. Subsequently, we found that the expression level of *ins-7* was significantly upregulated following celecoxib treatment in *pqm-1(* **−** *)* worms (Figure 4E, Table S2), but the degree of upregulation was lower than that of N2 worms. And, *pqm-1* expression level in *daf-16 (* **−** *)* worms was not significantly changed by10 µM celecoxib (Table S2). These results suggested that celecoxib positively regulated *ins-7* and that this was partially dependent on PQM-1.

### *ins-7* Expression in the Intestine is Upregulated by Celecoxib in *daf-16 (RNAi)* Worms

**Figure 5:**
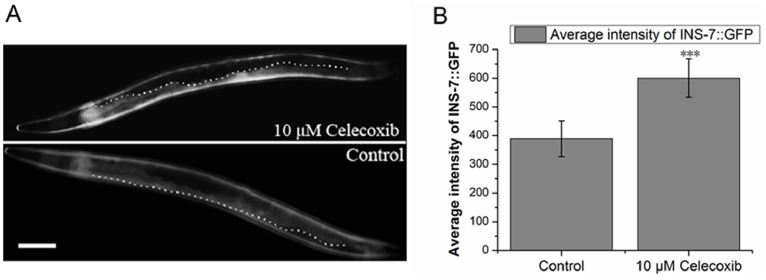
*ins-7::gfp* is regulated by celecoxib in *daf-16 (RNAi)* worms. *ins-7::gfp* (GFP flourescence below the dotted line, celecoxib-treated worm; above the dotted line, control worm) was significantly upregulated in *daf-16 (RNAi)* worms when treated with 10 µM celecoxib (**A**). The white bar represents 100 µM. The average flourencent intensity of INS-7::GFP was calculated using the Metamorph software package (Molecular Devices, Sunnyvale, CA, USA) (**B**). Figure B shows the mean average flourencent intensity of about 20 worms. Data are presented as mean ± SD. Error bars represent SD. P values were calculated using a T-test. ***P<0.001. Also see Table S5.

To further confirm our speculation, we performed RNAi knockdown of *daf-16* in an *ins-7::gfp* strain to test whether *ins-7* expression was regulated in *daf-16* null worms following celecoxib treatment. Eggs isolated from synchronous HT1702 worms were cultured on fresh RNAi plates containing bacterial strain HT115 expressing a double-stranded *daf-16* RNA and allowed to grow at 15°C; 3 days later, L4 stage nematodes were transferred to new plates seeded with the same bacteria in the presence or absence of 10 µM celecoxib and switched to 20°C for 24 hours. We used a fluorescent microscope to analysis *ins-7::gfp* expression. Our results were consistent with the previous reports indicating that *ins-7* could be expressed both in neurons and intestine cells (Figure 5A, Table S5) and indicated that the relative intensity of INS-7::GFP in the intestines of celecoxib-treated *daf-16 (RNAi)* worms was significantly higher than in the controls (Figure 5A and B, Table S5). This test confirmed our speculation that *ins-7* expression was positively regulated by other mechanisms independent of the DAF-16/IIS signal pathway.

## Discussion

A number of compounds and plant extracts of have been reported to influence aging in *C. elegans* [18], and pharmacological approaches to investigate aging are ongoing. A well-studied compound can be used to improve health and could also be very helpful for investigating the mechanisms of aging. Some compounds have been reported to extend the lifespan of *C. elegans* by regulating DAF-16 in the IIS signaling pathway. Celecoxib is a well-studied molecule that extends the lifespan of *C. elegans* by decreasing IIS signal transduction required to activate DAF-16 [19]. We noticed that this compound could not extend the lifespan of *daf-2* mutant worms, so we speculated that celecoxib could also influence DAF-2 ligands in *C. elegans*. In addition, when we tested the expression of DAF-16 target genes in celecoxib-treated worms and found that *ins-7* was abnormally upregulated by celecoxib, which conflicted with the idea that *ins-7* was downregulated when DAF-16 was activated or IIS signal transduction was decreased. This discrepancy led us to investigate the expression of *ins-7* in *C. elegans.*

In *C. elegans*, most insulin-like genes are expressed in the nervous system [12]. There are reports that *ins-7* is specifically expressed in nervous cells, but recently Murphy *et al.* [16] and Chen *et al.* [17] have reported that *ins-7::gfp* is also expressed in the intestine cells of *C. elegans*. Our results confirmed that *ins-7* is expressed in the intestine using the transgenic strain HT1702. *ins-7* expression was reported to be regulated by DAF-16 and DAF-2 in the intestine and to be downregulated in *daf-2(* **−** *)* worms [6]. We found that *ins-7* expression levels were upregulated in *daf-16 (* **−** *)* worms when treated with celecoxib and that the *ins-7::gfp* expression in the intestines of celecoxib-treated *daf-16 (RNAi)* worms was higher than in controls. According to these results, we speculated that *ins-7* expression was likely upregulated by other DAF-16–independent mechanisms. Additionally, we found that *ins-7* expression levels were upregulated to a lower but significant level in *daf-2;daf-16* double mutants when compared to *daf-16* mutants when both strains were treated with celecoxib. We speculated that the celecoxib upregulated *ins-7* expression levels were partially dependent on DAF-2 or that *ins-7* could regulate IIS activity via its feedback regulation of DAF-2 [16]. Our results also indicated that the *ins-7* gene expression in celecoxib-treated *daf-2 (* **−** *)* worms was significantly downregulated compared to *daf-2 (* **−** *)* control worms. This result was consistent with previous reports indicating that celecoxib negatively regulates IIS activity by decreasing PDK-1 activity [19]. Because PDK-1 is a downstream kinase in the IIS signaling pathway [31], *ins-7* expression levels could be downregulated by DAF-16 in *daf-2 (* **−** *)* worms when treated with celecoxib. However, according to our results, *ins-7* expression was significantly upregulated in celecoxib-treated N2 worms and downregulated in celecoxib-treated *daf-2 (* **−** *)* worms. Thus, the relationship between *ins-7* gene expression and DAF-2 function appeared to be interdependent (at least under celecoxib intervention) or, alternatively, the effect of celecoxib on *ins-7* expression could be altered by DAF-16 function in *daf-2 (* **−** *)* worms. However, these possibilities have yet to be confirmed. As such, our analysis showed that the expression level of *ins-7* in the intestine of *C. elegans* was negatively regulated by DAF-16 activity and could also be concurrently regulated in a positive manner by other mechanisms, at least following external pharmacological intervention.

DAF-16 directly regulates class I genes, but it may require some co-factors to regulate class II target genes. Tepper *et al.* reported that PQM-1 activates class II target gene transcription via DAE motifs at the posttranslational level [30]. Our results showed that celecoxib could also upregulate *ins-7* expression in *pqm-1* mutants, and we found that celecoxib could not enhance the PQM-1::GFP nuclear translocation in N2 worms. When we treated UL1735; *daf-16 (RNAi)* worms with celecoxib, we found that PQM-1::GFP nuclear localization was further increased. We speculated that there might be two possible reasons for this: first, the effect of celecoxib on PQM-1::GFP nuclear localization might be negated by a role for DAF-16 in the localization of PQM-1. Alternatively, the effect of celecoxib on PQM-1::GFP nuclear localization in otherwise wild-type worms might simply be below the threshold of our detection methods. However, *pqm-1* mutants did not completely repress the effect of celecoxib on *ins-7* expression, and this was also consisted with our speculation that *ins-7* was partially regulated by IIS and DAF-16.

In intestine cells, INS-7 can function as an agonist of DAF-2 and is negatively regulated by DAF-16 activity [6], [16]. *ins-7 (RNAi)* worms have a longer lifespan than wild-type worms, and *ins-7* expression in intestine cells of N2 worms is regulated by DAF-16 and the IIS signaling pathway [16]. We found that the expression level of *ins-7* in intestine cells of celecoxib-treated *daf-16 (RNAi)* worms was higher than in controls, indicating that *ins-7* expression could also be regulated by other mechanisms. Additionally, we found that the mean lifespan of *ins-7* mutant worms treated with celecoxib could be further increased compared to celecoxib alone. As such, we speculated that the lifespan-decreasing function of high *ins-7* expression levels in celecoxib-treated N2 worms might be suppressed or neutralized by the interaction of multiple mechanisms and the function of the IIS signaling pathway. As to whether or not celecoxib also regulates other anti-aging mechanisms in addition to the IIS signaling pathway has not been confirmed. However, the function of *ins-7* in nerve cells conflicts with that proposed for intestine cells. Chen *et al.* [17] reported that INS-7 in nerve cells can function as an antagonist of DAF-2 to activate DAF-16 activity. Recently, Ritter *et al.* [32] used 40 *C. elegans* insulin genes to study the balance between specificity and redundancy. They reported that no single insulin mutation or deletion completely recapitulated the phenotypes associated with perturbation of *daf-2* and that no single insulin gene was the sole agonist or antagonist for coordinating dauer formation via the DAF-2 receptor. As such, we feel that a more detailed investigation into the expression and function of insulin-like peptides in *C. elegans* is still required.

There are about 40 INS peptides that are reported to be ligands of DAF-2 in *C. elegans*, but the functions of many of these are not clear. Some studies have reported that *ins-1*, *ins-4, ins-5*, *ins-17*, *ins-18*, *ins-28*, and *ins-30* might have a function in the aging of *C. elegans* [33]–[36]. As such, we also tested if the expression of these genes could be influenced by celecoxib. We found there were no significant changes in *ins-1*, *ins-4*, *ins-5*, *ins-17*, *ins-18*, *ins-28*, or *ins-30* expression levels in celecoxib-treated N2 worms (data not show). There is, however, speculation that 2 or more INS peptides might combine to form a complex and that different INS peptides might combine to regulate aging, disease, and development of *C. elegans*. These different sets of INS peptides could have different functions in the recognition and response to many environmental stimuli [32]. Future studies will be needed to illuminate the expression patterns and functions of INS peptides in worms when they are under environmental or pharmacological stimuli or at different normal developmental stages. Our study confirmed that celecoxib extended the lifespan of *C. elegans* by decreasing IIS signaling and indicated that *ins-7* expression in N2 worms treated with celecoxib was abnormally upregulated. Together, our results suggested that the expression level of *ins-7*, a DAF-16 target gene, was negatively regulated by decreased IIS signal transduction and was positively regulated by other mechanisms when IIS signal transduction was decreased by pharmacological intervention. Accordingly, we suggest that *ins-7* is not an ideal reporter gene for DAF-16 activity and that the functions and regulated expression of INS peptides still require detailed study.

## Supporting Information

Table S1 **The expression profiles of DAF-16 target genes:** ***scl-20*** **,** ***K09F6.6*** **,** ***sod-3,*** **and** ***ins-7*** **in** ***daf-2*** **and** ***daf-16*** **mutants.** Young adult day 1 worms were used in these tests. The relative expression levels of the genes were determined using the 2^−ΔΔCT^ method and normalized to *cdc-42* and *act-1.*
(DOCX)

Table S2 **The expression profiles of DAF-16 target genes when N2,** ***daf-16 (*** **−** ***)*** **or** ***pqm-1(*** **−** ***)*** **worms were treated with 10 µM celecoxib.** The relative expression levels of the genes were determined using the 2^−ΔΔCT^ method and normalized to *cdc-42* and *act-1.*
(DOCX)

Table S3 ***ins-7*** **expression levels in** ***daf-2*** **and** ***daf-16*** **mutants when treated with celecoxib.** Young adult day 1 worms were transferred on to celecoxib-contained plates, and cultured for 24 hours at 20°C. The relative expression levels of the genes were determined using the 2^−ΔΔCT^ method and normalized to *cdc-42* and *act-1.*
(DOCX)

Table S4 **Nuclear translocation of DAF-16::GFP and PQM-1::GFP.** %: the percentage of worms demonstrating GFP nuclear localization. N: the number of worms that were analyzed in one experiment. n: the number of worms that demonstrated GFP nuclear localization.
(DOCX)

Table S5 **Average INS-7::GFP intensity in N2 worms treated with celecoxib is higher than that of controls.** The induction of the INS-7::GFP in the body of worms was assayed based on fluorescence using a Ti microscope (Nikon, Tokyo, Japan). The average GFP intensity was calculated by using the Metamorph software package (Molecular Devices, Sunnyvale, CA, USA).
(DOCX)

Table S6 **Primer sequences.**
(DOC)
