# Supplementary material for: ins-7 Gene Expression Is Partially Regulated by the DAF-16/IIS Signaling Pathway in Caenorhabditis elegans under Celecoxib Intervention
Source: PLoS One. 2014 Jun 19;9(6):e100320. doi: 10.1371/journal.pone.0100320 (PMC4063773; doi:10.1371/journal.pone.0100320)
Supplement: Table S1 — The expression profiles of DAF-16 target genes: scl-20 , K09F6.6 , sod-3, and ins-7 in daf-2 and daf-16 mutants. Young adult day 1 worms were used in these tests. The relative expression levels of the genes were determined using the 2−ΔΔCT method and normalized to cdc-42 and act-1. (DOCX) [file pone.0100320.s001.docx]

**Table S1.** **The expression profiles of DAF-16 target genes: *scl-20*, *K09F6.6*, *sod-3,* and *ins-7* in *daf-2* and *daf-16* mutants.**

| **Worm** | **Gene** | **Relative expression fold (vs N2)** | | | | | | **Average** | **SD** | **P value (t-test)** |
| --- | --- | --- | --- | --- | --- | --- | --- | --- | --- | --- |
|  |  | Exp1 | Exp 2 | Exp 3 | Exp 4 | Exp 5 | Exp 6 |  |  |  |
| ***daf-2(e1370)Ⅲ; daf-16 (mu86)Ⅰ*** | *ins-7* | 3.34 | 3.12 | 3.12 | 4.89 | 3.12 | 4.85 | 3.74 | 0.88 | <0.001 |
|  | *sod-3* | 0.95 | 0.96 | 0.92 | 0.88 | 1.02 | 0.96 | 0.94 | 0.04 | 0.32 |
| ***daf-2 (e1368) Ⅲ*** | *ins-7* | 0.37 | 0.83 | 0.58 | 0.24 | 0.42 | 0.39 | 0.47 | 0.20 | <0.001 |
|  | *sod-3* | 11.3 | 9.35 | 12.32 | 10.55 | 9.68 | 10.61 | 10.64 | 1.08 | <0.001 |
| ***daf-16 (mu86)Ⅰ*** | *ins-7* | 4.78 | 5.17 | 4.95 | 4.11 | 4.54 | 4.21 | 4.62 | 0.42 | <0.001 |
|  | *sod-3* | 1.16 | 1.02 | 1.18 | 1.03 | 1.04 | 0.98 | 1.06 | 0.08 | 0.36 |
| ***daf-2 (e1370)Ⅲ*** | *ins-7* | 0.19 | 0.22 | 0.21 | 0.21 | 0.29 | 0.21 | 0.22 | 0.03 | <0.001 |
|  | *sod-3* | 15.8 | 18.18 | 18.73 | 14.94 | 15.98 | 16.95 | 16.76 | 1.46 | <0.001 |
|  | *K09F6.6* | 0.32 | 0.54 | 0.46 |  |  |  | 0.44 | 0.11 | <0.001 |
|  | *scl-20* | 2.28 | 3.18 | 2.73 |  |  |  | 2.73 | 0.45 | <0.001 |

Young adult day 1 worms were used in these tests. The relative expression levels of the genes were determined using the 2^-△△CT^ method and normalized to *cdc-42* and *act-1.*
