# Supplementary material for: ins-7 Gene Expression Is Partially Regulated by the DAF-16/IIS Signaling Pathway in Caenorhabditis elegans under Celecoxib Intervention
Source: PLoS One. 2014 Jun 19;9(6):e100320. doi: 10.1371/journal.pone.0100320 (PMC4063773; doi:10.1371/journal.pone.0100320)
Supplement: Table S2 — The expression profiles of DAF-16 target genes when N2, daf-16 ( − ) or pqm-1( − ) worms were treated with 10 µM celecoxib. The relative expression levels of the genes were determined using the 2−ΔΔCT method and normalized to cdc-42 and act-1. (DOCX) [file pone.0100320.s002.docx]

**Table S2.** **The expression profiles of DAF-16 target genes when N2, *daf-16(-)* or *pqm-1(-)* worms were treated with 10 μM celecoxib.**

| **Worm** | **Gene** | **Relative expression fold (vs Control)** | | | **Average** | **SD** | **P value (t-test)** |
| --- | --- | --- | --- | --- | --- | --- | --- |
|  |  | Exp1 | Exp 2 | Exp 3 |  |  |  |
| **N2 (10 μM celecoxib), 24 hours** | *ins-7* | 3.8 | 4.43 | 4.21 | 4.14 | 0.32 | <0.001 |
|  | *sod-3* | 2.65 | 2.72 | 2.6 | 2.66 | 0.06 | <0.001 |
|  | *scl-20* | 1.75 | 1.63 | 1.81 | 1.73 | 0.09 | <0.05 |
|  | *K09F6.6* | 0.49 | 0.37 | 0.64 | 0.5 | 0.14 | <0.01 |
|  |  |  |  |  |  |  |  |
| **N2 (10 μM celecoxib), 7 days** | *ins-7* | 2.56 | 3.13 | 2.76 | 2.82 | 0.28 | <0.001 |
|  | *sod-3* | 2.03 | 1.95 | 2.13 | 2.04 | 0.09 | <0.001 |
|  | *scl-20* | 1.52 | 1.61 | 1.38 | 1.5 | 0.12 | <0.05 |
|  | *K09F6.6* | 0.61 | 0.58 | 0.73 | 0.64 | 0.08 | <0.05 |
| **N2 (10 μM celecoxib), 14 days** | *ins-7* | 1.59 | 2.01 | 1.84 | 1.81 | 0.21 | <0.01 |
|  | *sod-3* | 1.35 | 1.56 | 1.62 | 1.51 | 0.14 | <0.05 |
|  | *scl-20* | 1.25 | 1.36 | 1.45 | 1.35 | 0.1 | <0.05 |
|  | *K09F6.6* | 0.89 | 0.91 | 0.75 | 0.85 | 0.08 | >0.05 |
| ***pqm-1(-)* (10 μM celecoxib), 24 hours** | *ins-7* | 2.12 | 2.43 | 2.44 | 2.33 | 0.18 | <0.001 |
| ***daf-16(-)* (10 μM celecoxib), 24 hours** | *pqm-1* | 0.95 | 1.11 | 1.06 | 1.04 | 0.08 | >0.05 |

The relative expression levels of the genes were determined using the 2^-△△CT^ method and normalized to *cdc-42* and *act-1.*
