# Supplementary material for: ins-7 Gene Expression Is Partially Regulated by the DAF-16/IIS Signaling Pathway in Caenorhabditis elegans under Celecoxib Intervention
Source: PLoS One. 2014 Jun 19;9(6):e100320. doi: 10.1371/journal.pone.0100320 (PMC4063773; doi:10.1371/journal.pone.0100320)
Supplement: Table S3 — ins-7 expression levels in daf-2 and daf-16 mutants when treated with celecoxib. Young adult day 1 worms were transferred on to celecoxib-contained plates, and cultured for 24 hours at 20°C. The relative expression levels of the genes were determined using the 2−ΔΔCT method and normalized to cdc-42 and act-1. (DOCX) [file pone.0100320.s003.docx]

**Table S3.** ***ins-7* expression levels in *daf-2* and *daf-16* mutants when treated with celecoxib.**

| **Worms** | **Relative expression fold (vs control)** | | | **Average** | **SD** | **P value (t-test)** |
| --- | --- | --- | --- | --- | --- | --- |
|  | Exp 1 | Exp 2 | Exp3 |  |  |  |
| ***daf-2 (e1370)Ⅲ*** | 0.46 | 0.69 | 0.54 | 0.56 | 0.09 | 0.009 |
| ***daf-16 (mu86)Ⅰ*** | 3.56 | 4.79 | 4.04 | 4.13 | 0.51 | 0.001 |
| ***daf-2(e1370)Ⅲ; daf-16 (mu86)Ⅰ*** | 1.79 | 1.59 | 1.85 | 1.74 | 0.14 | 0.019 |
| ***daf-2 (e1368) Ⅲ*** | 0.41 | 0.48 | 0.44 | 0.44 | 0.02 | 0.0002 |

Young adult day 1 worms were transferred on to celecoxib-contained plates, and cultured for 24 hours at 20℃. The relative expression levels of the genes were determined using the 2^-△△CT^ method and normalized to *cdc-42* and *act-1.*
