# Supplementary material for: ins-7 Gene Expression Is Partially Regulated by the DAF-16/IIS Signaling Pathway in Caenorhabditis elegans under Celecoxib Intervention
Source: PLoS One. 2014 Jun 19;9(6):e100320. doi: 10.1371/journal.pone.0100320 (PMC4063773; doi:10.1371/journal.pone.0100320)
Supplement: Table S4 — Nuclear translocation of DAF-16::GFP and PQM-1::GFP. %: the percentage of worms demonstrating GFP nuclear localization. N: the number of worms that were analyzed in one experiment. n: the number of worms that demonstrated GFP nuclear localization. (DOCX) [file pone.0100320.s004.docx]

**Table S4. Nuclear translocation of DAF-16::GFP and PQM-1::GFP.**

|  | **DAF-16::GFP** | | **PQM-1::GFP** | | **PGM-1::GHP/daf-16 RNAi** | |
| --- | --- | --- | --- | --- | --- | --- |
|  | Control  %(n/N) | 10 μM Celecoxib  %(n/N) | Control  %(n/N) | 10 μM Celecoxib  %(n/N) | Control  %(n/N) | 10 μM Celecoxib  %(n/N) |
| **Experiment 1** | 7.2(9/125) | 42.7(44/103) | 40.4(40/99) | 33.9(42/124) | 42.0(50/119) | 66.0(70/106) |
| **Experiment 2** | 3.4(4/117) | 34.4(40/116) | 50.9(54/106) | 46.4(53/114) | 61.2(74/121) | 81.6(84/103) |
| **Experiment 3** | 12.2(12/98) | 25.6(31/121) | 60.9(64/105) | 51.9(54/104) | 56.6(69/122) | 73.9(88/119) |
| **Average** | 7.6 | 34.2 | 50.7 | 44 | 53.2 | 73 |
| **SD** | 4.4 | 8.5 | 10.2 | 9.2 | 10 | 7.8 |
| **t-test** |  | 0.008 |  | 0.449 |  | 0.048 |

%: the percentage of worms demonstrating GFP nuclear localization. N: the number of worms that were analyzed in one experiment. n: the number of worms that demonstrated GFP nuclear localization.
