# Supplementary material for: ins-7 Gene Expression Is Partially Regulated by the DAF-16/IIS Signaling Pathway in Caenorhabditis elegans under Celecoxib Intervention
Source: PLoS One. 2014 Jun 19;9(6):e100320. doi: 10.1371/journal.pone.0100320 (PMC4063773; doi:10.1371/journal.pone.0100320)
Supplement: Table S5 — Average INS-7::GFP intensity in N2 worms treated with celecoxib is higher than that of controls. The induction of the INS-7::GFP in the body of worms was assayed based on fluorescence using a Ti microscope (Nikon, Tokyo, Japan). The average GFP intensity was calculated by using the Metamorph software package (Molecular Devices, Sunnyvale, CA, USA). (DOCX) [file pone.0100320.s005.docx]

**Table S5. Average INS-7::GFP intensity in N2 worms treated with celecoxib is higher than that of controls.**

| **Worms** | **Control** | **10 μM Celecoxib** |
| --- | --- | --- |
| **1** | 391.40 | 693.26 |
| **2** | 546.00 | 672.61 |
| **3** | 309.95 | 574.20 |
| **4** | 531.38 | 732.64 |
| **5** | 332.55 | 606.08 |
| **6** | 316.26 | 688.85 |
| **7** | 388.67 | 514.92 |
| **8** | 365.70 | 578.47 |
| **9** | 351.20 | 574.96 |
| **10** | 459.65 | 613.50 |
| **11** | 431.05 | 588.77 |
| **12** | 396.46 | 517.99 |
| **13** | 431.12 | 511.34 |
| **14** | 342.71 | 559.31 |
| **15** | 355.16 | 578.18 |
| **16** | 361.53 | 626.08 |
| **17** | 343.49 | 530.31 |
| **18** | 387.10 | 527.76 |
| **19** | 383.84 | 714.03 |
| **20** | 356.67 | 598.06 |
| **Average** | 389.09 | 600.071 |
| **SD** | 62.21 | 66.812 |
| **T-test** |  | <0.001 |

The induction of the INS-7::GFP in the body of worms was assayed based on fluorescence using a Ti microscope (Nikon, Tokyo, Japan). The average GFP intensity was calculated by using the Metamorph software package (Molecular Devices, Sunnyvale, CA, USA).
