# Supplementary material for: ins-7 Gene Expression Is Partially Regulated by the DAF-16/IIS Signaling Pathway in Caenorhabditis elegans under Celecoxib Intervention
Source: PLoS One. 2014 Jun 19;9(6):e100320. doi: 10.1371/journal.pone.0100320 (PMC4063773; doi:10.1371/journal.pone.0100320)
Supplement: Table S6 — Primer sequences. (DOC) [file pone.0100320.s006.doc]

**Table S6. Primer sequences**

| *cdc-42F* | 5’- CTGCTGGACAGGAAGATTACG-3’ |
| --- | --- |
| *cdc-42R* | 5’- CTCGGACATTCTCGAATGAAG-3’ |
| *sod-3F* | 5’-AGCATCATGCCACCTACGTGA-3’ |
| *sod-3R* | 5’-CACCACCATTGAATTTCAGCG-3’ |
| *ins-7 F* | 5'-CATGCGAATCGAATACTGAA-3' |
| *ins-7 R* | 5'-CACTGTTTTCGAATGAAGTC-3' |
| *scl-20 F* | 5'-AACCACATTAAAAACACAATCCG-3' |
| *scl-20 R* | 5'-TACTCACTGCGAAAAACATCTGA-3' |
| *K09F6.6 F* | 5'-TGTCGTCATGCAAAGAAAGC-3' |
| *K09F6.6 R* | 5'-TTAGTTGGTTGGATGCCACA-3' |
| *act-1 F* | 5'-TCGGTATGGGACAGAAGGAC-3' |
| *act-1 R* | 5'-CATCCCAGTTGGTGACGATA-3'  GACGATA |
| *pqm-1 F* | 5'-ATGAAGCCATGGAAATCGAG-3' |
| *pqm-1 R* | 5'-TGGAGACAGCAGAAATGACG-3' |
